# Effects of Sampling Conditions and Environmental Factors on Fecal Volatile Organic Compound Analysis by an Electronic Nose Device

**DOI:** 10.3390/s16111967

**Published:** 2016-11-23

**Authors:** Daniel J. C. Berkhout, Marc A. Benninga, Ruby M. van Stein, Paul Brinkman, Hendrik J. Niemarkt, Nanne K. H. de Boer, Tim G. J. de Meij

**Affiliations:** 1 Department of Pediatric Gastroenterology, Emma Children’s Hospital/Academic Medical Center, Meibergdreef 9, 1105 AZ Amsterdam, The Netherlands; m.a.benninga@amc.uva.nl; 2 Department of Pediatric Gastroenterology, VU University Medical Center, De Boelelaan 1117, 1081 HV Amsterdam, The Netherlands; r.m.van.stein@student.vu.nl (R.M.v.S.); t.demeij@vumc.nl (T.G.J.d.M.); 3 Department of Respiratory Medicine, Academic Medical Center, Meibergdreef 9, 1105 AZ Amsterdam, The Netherlands; p.brinkman@amc.uva.nl; 4 Neonatal Intensive Care Unit, Máxima Medical Center, De Run 4600, 5504 DB Veldhoven, The Netherlands; Hendrik.Niemarkt@mmc.nl; 5 Department of Gastroenterology and Hepatology, VU University Medical Center, De Boelelaan 1117, 1081 HV Amsterdam, The Netherlands; KHN.deBoer@vumc.nl

**Keywords:** electronic nose, Cyranose320^®^, standardization, volatile organic compound, feces, sampling conditions, smell

## Abstract

Prior to implementation of volatile organic compound (VOC) analysis in clinical practice, substantial challenges, including methodological, biological and analytical difficulties are faced. The aim of this study was to evaluate the influence of several sampling conditions and environmental factors on fecal VOC profiles, analyzed by an electronic nose (eNose). Effects of fecal sample mass, water content, duration of storage at room temperature, fecal sample temperature, number of freeze–thaw cycles and effect of sampling method (rectal swabs vs. fecal samples) on VOC profiles were assessed by analysis of totally 725 fecal samples by means of an eNose (Cyranose320^®^). Furthermore, fecal VOC profiles of totally 1285 fecal samples from 71 infants born at three different hospitals were compared to assess the influence of center of origin on VOC outcome. We observed that all analyzed variables significantly influenced fecal VOC composition. It was feasible to capture a VOC profile using rectal swabs, although this differed significantly from fecal VOC profiles of similar subjects. In addition, 1285 fecal VOC-profiles could significantly be discriminated based on center of birth. In conclusion, standardization of methodology is necessary before fecal VOC analysis can live up to its potential as diagnostic tool in clinical practice.

## 1. Introduction

Volatile organic compounds (VOC) are carbon-based, organic chemicals that can be perceived by olfactory sense. Over the past decades, much effort has been carried out within the technical field of VOC detection. In 1982, an artificial nose imitating the mammalian olfactory system was introduced [1]. This instrument consisted of three different metal-oxide gas sensors and allowed for accurate discrimination between VOC profiles of complex gaseous mixtures [1]. Recently, major advances in nanotechnology, computer software, and material of sensors have facilitated the implementation of eNose devices in a variety of industries, including cosmetics, food, military, pharmaceutical and agricultural industry [2].

Human VOCs are produced during both physiologic and pathophysiologic metabolic processes and excreted as waste product through all conceivable bodily excrements, including sweat, feces, urine and exhaled breath [3]. Consequently, scent has long been appreciated as an interesting target for clinical diagnostics. In medicine, ancient traditional Chinese physicians were the first to use their olfactory organ as diagnostic tool [4]. Since then, a rapidly increasing number of studies have demonstrated the potential of VOC analysis as noninvasive diagnostic biomarker for malignancies, inflammatory, infectious and metabolic diseases [5,6,7,8,9,10,11,12].

Over the past years, remarkable advances in breathomics have been made as exhaled VOCs belong to the most extensively studied [13]. Since VOCs either have a local, systemic or exogenous origin [14], these exhaled VOC studies are not restricted to pulmonary diseases, making exhaled volatiles an area of growing clinical interest. The latest efforts in this field have focused on detection of potential confounding factors and optimization of the sampling and storing techniques [15,16,17,18]. Despite these attempts, standardization remains a critical issue hampering implementation of breath analysis into clinical practice [19].

Although less studied, fecal gas may be an interesting equivalent of exhaled breath. The fecal headspace harbors volatiles deriving from the unique interaction between host (patho) physiological metabolism, gut microbiome and the final pathways of nutrition and digestion [20]. Many available fecal VOC studies have exclusively focused on differences in VOC composition between diseased subjects and healthy controls. Research on the potential influence of sampling, storing and analytical conditions on fecal VOC outcome remains scarce. Furthermore, lack of standardization hampers reliable comparison between different study outcomes. Insight in effects of environmental factors on (fecal) VOC profiles is necessary to limit bias and to define optimal conditions for VOC analysis, prior to implementation in clinical practice.

The purpose of this study was to assess the effects of environmental factors, the influence of sampling conditions and influence of sampling method on fecal VOC composition, as measured by an eNose.

## 2. Materials and Method

Effects of the following variables on VOC composition were measured: fecal sample mass, number of freeze–thaw cycles, fecal sample temperature, water content, duration of storage at room temperature and sampling method (rectal swab vs. feces). Influence of center of origin on VOC profile was evaluated by comparing fecal VOCs from infants born at three different centers.

### 2.1. Sample Collection and Preparation

The influence of fecal sample mass, number of freeze–thaw cycles, fecal sample temperature, water content and duration of storage at room temperature on fecal VOC composition was performed by preparing subsamples taken from a fecal mass, which was constructed from multiple stool samples from one healthy donor. By stirring with a glass spatula for twenty minutes, this fecal mass was homogenized. Per experiment, fecal samples were acquired from this fecal mass, allowing for adequate comparison between samples. We compared the variables of interest with baseline characteristics, defined as undiluted fecal samples weighing 0.50 g, stored at −20 °C immediately after collection, and gradually heated in an incubator to 37 °C during one hour prior to VOC analysis. These characteristics have been used in previous studies on the potential of VOC profiling in the (early) detection of colorectal cancer, pediatric inflammatory bowel disease, necrotizing enterocolitis and late-onset sepsis, using the same eNose device as in the present study. Applying these baseline characteristics VOC analysis allowed for discrimination between these diseases and healthy controls [7,8,9,21]. Per experiment, only the variable of interest changed according to the protocol, all other variables retained the same, corresponding the baseline characteristics. Samples generated for the evaluation of fecal sample mass, number of freeze–thaw cycles, fecal sample temperature and water content were obtained in fiftyfold (i.e., for each variable of interest, 50 subsamples were taken from the homogenized fecal mass for VOC analysis).

Comparison between VOC profiles obtained from rectal swabs and fecal samples was performed by collecting fecal samples and rectal swabs (FLOQSwabs, Copan Flock Technologies, Brescia, Italy) from 25 healthy adults. Donors were instructed to collect the rectal swab five minutes after defecation by introducing the swab 1 centimeter past the rectal sphincter and subsequently rotate the swab 360°.

Unless stated otherwise, all obtained samples (fecal samples and swabs) were stored at −20 °C immediately after collection and transferred into a 3 mL sealed vacutainer (BD vacutainer, Franklin Lakes, NJ, USA) and subsequently placed in a 37 °C stove for one hour prior to analysis, enabling fecal VOCs to fill the headspace.

### 2.2. Variables of Interest

The influence of fecal sample mass on VOC profiles was analyzed by comparing fecal samples weighing 0.20 and 2.0 g mutually and with samples containing the baseline characteristics (*n* = 150 samples).

In order to study the effect of number of freeze–thaw cycles on the obtained VOC-profile, VOCs from fresh fecal samples, (analyzed directly after production, without being frozen) and samples undergoing three freeze (−20 °C )—thaw (until room temperature) cycles were compared mutually and with baseline samples (*n* = 150 samples).

The effect of sample temperature on fecal VOC profiles was assessed by comparing samples heated until 4 °C and 21 °C mutually and with samples containing the baseline characteristics (*n* = 150 samples).

VOCs from samples containing different degrees of water, including dilution 1:1, 1:2 and 1:5 (feces (g):H_2_O (mL)) were compared mutually and with baseline samples in order to assess influence of water content on the fecal VOC profile (*n* = 200). All diluted samples were homogenized by centrifugation prior to analysis.

Effect of duration of storage at room temperature was analyzed by comparing VOC profiles from 15 fresh fecal samples with VOC profiles from 15 samples kept at room temperature during 3 h (*n* = 30 samples).

VOC profiles from 25 rectal swabs were compared with VOC profiles from 25 fecal samples of the same subjects and also with VOCs deriving from 25 empty (non-used) swabs. The latter analysis was performed to assess whether it was feasible to obtain a rectal VOC profile (*n* = 75).

The influence of center of origin on VOC outcome was assessed by using VOC profiles obtained by a Cyranose320^®^ (Smiths Detections, Pasadena, CA, USA) in a previous study on fecal VOC profiling in the early detection of necrotizing enterocolitis and late-onset sepsis in neonates [9,21]. In that study, neonates born at a gestational age of <30 weeks admitted at the neonatal intensive care units (NICU) of VU medical center (VUmc), Academic Medical Center (AMC), both allocated in Amsterdam, and Maxima Medical Center (MMC) in Veldhoven were included. Totally 1285 fecal samples from 71 different infants (Table S1) were collected (Stuhlgefäß 10 mL, Frickenhausen, Germany) daily from the diaper, from birth until a postnatal age of 28 days. Since characteristics of patients included in each center were comparable, including gestational age and birth weight, the influence of center-bound environmental factors on fecal VOC profiles linked to local differences in medical protocols, including choice of antibiotics and formula feeding, could be assessed.

### 2.3. VOC Analysis 

All samples were analyzed in random order with exception of the fifty fresh samples. Two needles (BD blunt fill needle 1.2 mm × 40 mm, Franklin Lakes, NJ, USA) were pierced into the cap of the vacutainer and connected by a tube (Argyle Covidien tube 3 mm, Mansfield, MA, USA) to the Cyranose320^®^ (Smiths Detections, Pasadena, CA, USA) in an air-tight loop system, preventing dilution with ambient air. A baseline reference signal was obtained during 30 s prior to each measurement by connecting a VOC-filter (A1, North Safety, Middelburg, The Netherlands) to the inlet of the eNose. The actual measurement was performed by letting the fecal VOCs in the headspace pass an array of 32 sensors for 60 s (a schematic illustration of the measurement setup is depicted in Figure S1).

### 2.4. Cyranose320^®^

Each eNose sensor comprises a unique non-conducting polymer coating, containing conductive carbon black material [22]. The polymer coatings swell upon contact with presented VOCs, leading to an increase in electrical resistance by augmenting the distance between the conductive particles [22]. Each individual VOC interacts with multiple sensors and each individual sensor interacts with multiple VOCs, leading to 32 unique electrical resistance changes, resulting in a so-called “smell-print” of the presented gaseous mixture [22]. These individual smell-prints can be compared based on pattern recognition algorithms. Sensors were purged for 90 s after each measurement to remove any remaining fecal VOCs from the sensors.

### 2.5. Data Analysis

VOC-profiles were analyzed with Statistical Package for the Social Science software version 22 (SPSS Inc., Chicago, IL, USA). Variance of the raw data from the 32 sensors was recombined in a set of four principle components (PC) by principle component analysis per experiment. Principal components (PC) differing thrice the standard deviation or more from the mean were interpreted as potential outliers and consequently were excluded from further analysis. One-Way ANOVA tests were performed on all variables except “duration of storage at room temperature”, on which an independent *t*-test was performed to assess discriminating PCs. A *p*-value of <0.05 was considered statistically significant. Selected PCs were used in a canonical discriminant analysis (CDA), before internally cross-validated by means of leave one out method. Per variable of interest, overall accuracy with corresponding sensitivity and specificity values are provided. Scatter plots for the discrimination between samples were created for each variable of interest. Axes depict two orthogonal linear recombinations of the raw sensor data by means of PC analysis; individual VOC profiles are illustrated as marked points. The intersection of the lines deriving from the individual profiles demonstrates the mean VOC profile of this specific variable of interest.

## 3. Results 

Number of PCs differing ≥3 standard deviation from the mean and consequently were excluded from further analysis are depicted in Table S2.

Fecal samples weighing 2.0 g demonstrated a significant different VOC profile compared to fecal samples with a mass of 0.2 g and 0.5 g (PC1 and PC2: *p*-value < 0.001 in both cases). Likewise, VOC profiles from samples with a mass of 0.5 g differed significantly from 0.2 g samples (PC3: *p*-value = 0.016) (Table 1). Sample mass can be discriminated based on fecal VOC profiles with an overall accuracy of 64.4% and corresponding sensitivity and specificity levels ranging from 52.7%–81.6% and 55.1%–80.0% respectively (Table 2).

Baseline samples enduring one freeze–thaw cycle demonstrated a significant difference in all four PCs when compared to both fresh samples as well as samples enduring three cycles. Three PCs differed significantly when fresh fecal samples were compared with samples enduring three freeze–thaw cycles (PC2, PC3 and PC4 with *p*-values of 0.001, <0.001 and <0.001, respectively) (Table 1). Fecal VOC profiles from samples with varying freeze–thaw cycles can be discriminated with an overall accuracy of 89.7% with corresponding sensitivity and specificity values ranging from 83.6%–100.0% and 82.0%–95.7%, respectively (Table 2).

Samples analyzed at different temperatures provided significant different fecal VOC profiles with PC1 *p*-values being either equal to 0.001 (21 °C vs. 37 °C) or less than 0.001 (both 4 °C vs. 21 °C and 4 °C vs. 37 °C) (Table 1). Discrimination between fecal samples with varying temperatures based on fecal VOC profiles was possible with an accuracy of 78.4% and corresponding sensitivity and specificity values ranging between 66.0%–100.0% and 70.0%–93.9%, respectively (Table 2).

Varying water content of the fecal samples significantly altered measured fecal VOC profile composition. Fecal VOC profiles from samples containing the lowest water content (1:0) differed from samples diluted on a scale of 1:1 (PC2: *p*-value = 0.017). Mutual comparison of all other varying water contents differed significantly with corresponding *p*-values of either equal to, or less than 0.002 (1:0 vs. 1:2, 1:0 vs. 1:5, 1:1 vs. 1:2 and 1:1 vs. 1:5 with corresponding *p*-values of <0.001, <0.001, 0.001, and 0.002, respectively). Only exception is further dilution from 1:2 to 1:5, since this did not cause a significant shift in VOC profile (PC 4: *p*-value = 0.643). Electronic nose analysis allowed for discrimination of fecal samples with varying water content with an overall accuracy of 38.1% (Table 2).

**Figure 1:**
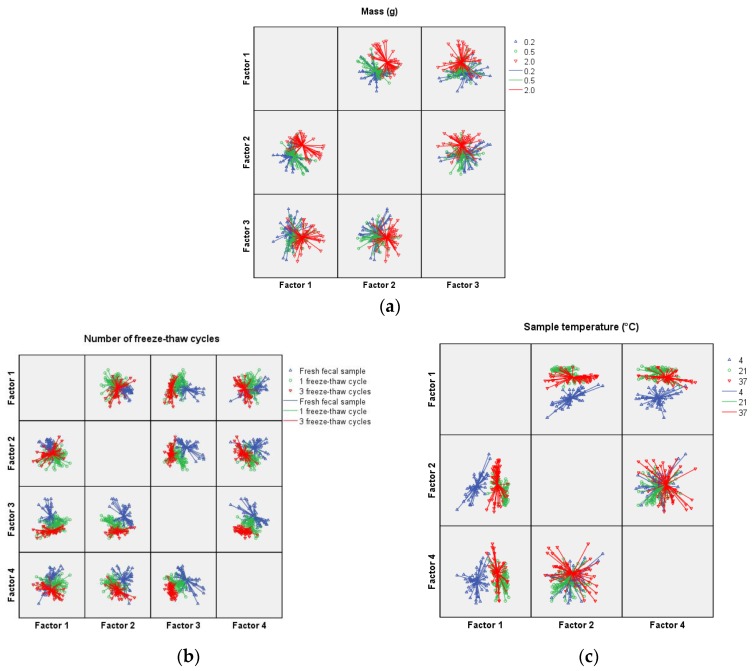

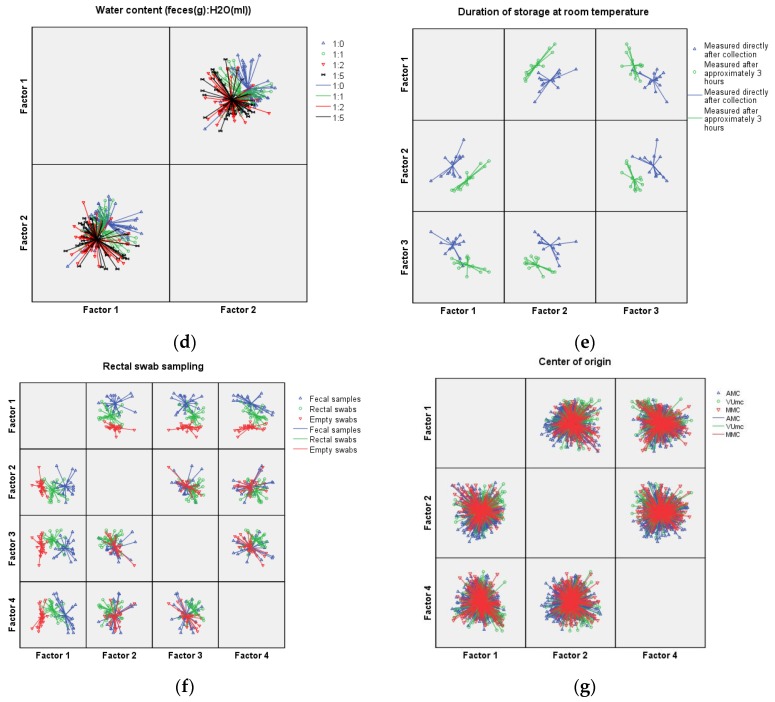
(**a**–**g**) Scatterplot for the discrimination by electronic nose based on difference in several variables, including: (**a**) sample mass; (**b**) number of freeze–thaw cycles; (**c**) sample temperature; (**d**) water content; (**e**) duration of storage at room temperature; (**f**) rectal swabbing; and (**g**) center of origin. Axes depicted are orthogonal linear recombinations of the raw sensor data by means of principle component analysis. Illustrated axes solely comprise principle components demonstrated to be statistically significant different for the variable concerned. Individual VOC profiles are illustrated as marked points. The intersection of the lines deriving from the individual profiles demonstrates the mean VOC profile of this specific variable. All evaluated sampling conditions have a significant influence on detected fecal VOC profile, only dilution from 1:2 to 1:5 did not affect outcome. Abbreviations: AMC = Academic medical center; MMC = Maxima Medical Center; VUmc = Vrije Universiteit medical center.

Fecal VOC profiles from fecal samples measured directly after production exhibit a significant different VOC profile compared to samples stored at room temperature for approximately 3 h (PC1, PC2 and PC 3: *p*-value ≤ 0.001) (Table 1). By depicting these two orthogonal linear recombinations of the raw sensor data by means of principle component analysis, this difference can be well visualized (Figure 1e). Fecal samples analyzed directly after production can be discriminated from fecal samples stored at room temperature with an accuracy of 100% (Table 2).

Significant differences in VOC profiles were observed between VOC profiles obtained from fecal samples, rectal swabs and empty swabs (PC 1: *p*-value < 0.001) with corresponding sensitivity and specificity values of 78.9% and 68.2%, 66.7% and 75.0%, and 92.0% and 92.0%, respectively (Table 1 and Table 2).

VOC-profiles (*n* = 1285) from 71 infants, clustered based on center, could significantly be discriminated based on center of origin (AMC vs. VUmc, AMC vs. MMC and VUmc vs. MMC with corresponding *p*-values 0.003, <0.001 and 0.031 respectively) with an overall accuracy of 39.6%, results are shown in Table 1 and Table 2, and Figure 1g.

## 4. Discussion

In the present study, we observed that several environmental factors and sampling conditions significantly influenced fecal VOC profiles. In addition, VOC profiles obtained by sampling with a rectal swab differed from VOC profiles of fecal samples.

Thus far, few studies have investigated optimal sampling conditions for fecal VOC analysis, mostly using the chemical analytical technique “gas chromatography-mass spectrometry” (GC-MS) [23,24,25,26,27]. GC-MS allows for identification of individual VOCs, based on their physiochemical properties, whereas an eNose harbors a chemical array sensor system, allowing for identification of complex gaseous mixtures by deploying pattern recognition algorithms [3,28]. In eNose devices, the physical quantity of each individual sensor, such as electrical resistance or oscillation frequency, can be influenced by the entire spectrum of VOCs present in a gaseous mixture [3]. Confounding VOCs, originating from non-relevant or non-avoidable environmental, host-specific or sampling-specific sources, may predominantly interact with these nanosensors, instead of those VOCs of potential interest [29,30]. Contrariwise, whereas a single VOC alteration based on a given confounder may cause significant changes in GC-MS results, this could have negligible effect on the array of cross-reactive sensors in an eNose [15]. As a result, optimal sampling and storing conditions for chemical analytical techniques may differ from optimal conditions in VOC profiling by eNose. In addition to chemical analytical techniques, studies on optimal sampling conditions regarding intestinal microbiota analysis may provide useful guidance, since fecal VOCs are largely generated during microbial metabolic processes and thus considered to reflect microbiota composition [31]. Notably, recent meta-analysis demonstrated gut microbiota to cluster primarily by study, illustrating the common necessity to standardize sampling and analytical conditions for both microbiota and VOC analysis [32]. Storing and sampling conditions impairing gut microbiome preservation potentially have a significant influence on fecal VOC composition as well. The significant effect of the microbiome on the volatolome is well illustrated in a recently published study demonstrating that interventions with a significant effect on the gut microbiome, such as bowel cleansing and administration of antibiotics in Helicobacter pylori eradication therapy, have a significant effect on detected volatiles [33]. In addition, fecal VOC profiles from patients with a gastrointestinal *Clostridium difficile* infection exhibit a significant different volatile profile compared to non-infected controls [34,35,36]. Both examples emphasize the impact of gut microbiome on fecal volatolome. However, since fecal VOCs also originate from other sources than intestinal microbiota, this could hamper translation from outcomes of studies on effects of environmental factors on microbiota composition to effects on fecal VOC profiles.

In the following sections, results per variable will be discussed and compared to available literature.

Consistent with results from fecal volatile studies using GC-MS, we measured significant differences in VOC profiles between samples weighing 0.20 g and 0.50 g [23,24]. Reade and colleagues demonstrated eight compounds to be detectable at significant higher intensities in samples weighing 450 mg compared to samples with a mass of 100 mg (2,3-butanedione, tetrahydrofurane, ethyl ester propanoic acid, n-propyl acetate, 2-pentenal E, propyl ester propanoic acid, 2-methylpropanal and 1-propanol) [16]. In contrast, results of fecal VOC profile comparison between samples containing higher masses are ambiguous [23,24,26]. Reade and colleagues state in their manuscript that the applied solid phase micro-extraction (SPME) fiber, required for capturing fecal VOCs prior to analysis, reached its limit of absorbance at 0.45 g [24]. However, this detected amount and concentration of fecal VOCs by GC-MS is dependent on the applied type of SPME filter.

Observed differences in VOC profiles between fresh samples and fecal samples enduring one or multiple freeze–thaw cycles are in line with results using a different eNose (Aetholab^®^, The eNose Company, Zutphen, The Netherlands) [20,37]. Microbial integrity seems to be preserved when fecal samples are rapidly frozen after defecation but it does influence the metabolic activity [20,38]. Probably, production of metabolic co-products by intestinal microbes are halted during immediate freezing and do not recover completely upon thawing. However, recent fecal metabolomics studies demonstrated a significant increase in relative concentration of several branched-chain and aromatic amino acids in samples enduring one or multiple freeze–thaw cycles (both −20 °C and −80 °C) compared to fresh samples, suggesting a release of microbial intracellular contents following freeze–thaw cycles [27,39]. Notably, using GC-MS, similar fecal VOC profiles were detected deriving from fecal samples stored at −20 °C for seven days, compared to fresh samples, although, unfortunately, additional data was not published [40]. It remains to be elucidated to what extent fecal VOCs are influenced by the duration of being in frozen state. For urine samples stored at −80 °C, the shelf life, based on VOC concentration and diversity, appears to be nine months [41]. Future research needs to assess the influence of duration of freezing on fecal volatiles analyzed by means of an eNose.

High temperatures lead to release of more different types of volatiles at increased concentrations compared to low temperatures, consequently influencing VOC composition [39,42]. This is probably the reason for the observed differences in VOC profiles measured at different temperatures in the present study. With higher sample temperature, more organic compounds become volatile with higher intensities. An additional explanation might be that microbial fermentation processes are increased at higher temperatures [39]. Since different VOCs have specific optimal temperatures to be in volatile state, optimal temperature depends on VOC of interest, which may differ for different (clinical) applications.

In the current study, VOC profiles from samples containing the two highest water contents did not differ significantly from each other, as opposed to other studied dilutions. Clinically, this detected difference is of importance since several gastrointestinal diseases are associated with diarrhea and thus with high water output, diluting feces. Presumably, fecal VOCs present in the headspace are also diluted by the added water, leading to a change in detected VOC profile. Eventually fecal VOCs are outnumbered in samples containing a high degree of water content, resulting in highly diluted fecal VOCs that provide a signal too low for the eNose sensors to discriminate between these samples. In these cases, VOCs deriving from the added water have the upper hand. Although statistically significant, performance characteristics after internal validation are suboptimal. This can partly be explained by the non-significant difference between samples with the two highest water contents. However, if fecal VOC analysis can be used in clinical setting remains to be elucidated by comparing samples, containing varying water contents, from healthy and diseased. Lyophilization may offer a solution to this problem, however freeze-drying may reduce the content of volatiles, thereby hampering adequate comparison between samples [27,43].

Fecal VOC profiles significantly altered when kept at room temperature for approximately three hours, compared to a immediately after production with an accuracy of 100%. Similar results were obtained in a study about fecal metabolite preservation [39]. Fecal samples were found to be highly unstable over time when left at room temperature, mainly due to ongoing fermentative processes, resulting in distinct metabolic profiles based on time-dependent differences between samples [39]. No significant change in VOC abundances was demonstrated in GC-MS analysis when samples were left at 1 °C for a period of 14 h [24]. This incongruence can be explained by the difference in sample temperature between the studies, since fermentative processes are reduced at lower temperatures [39]. Another study compared VOC profiles deriving from fresh blood samples with those of blood samples stored at room temperature for a period of six weeks, as measured by GC-MS. Significant differences in VOC profiles were observed over time, characterized by a wider range of chemical classes detected, increasing VOC complexity [44]. Whereas 1-octen-3-ol represents the only compound detected immediately after blood collection, degraded blood samples contained, besides 1-octen-3-ol, several other compounds, including 2-heptatone, 4-heptatone 2-ethyl-1-hexanol and 2-pentylfuran [29]. In addition, breath samples are subject to a decreased stability of several compounds within hours following collection in a sampling bag. Pronounced losses of mainly high molecular mass volatiles were demonstrated. Apart from background emission of contaminants and release of compounds from the bag, diffusion of volatiles through the sampling bag wall also contributed to variation in detected compounds [19,45]. Similar processes may distort adequate comparison between fresh fecal samples and samples kept at room temperature for a specific time. Ideally, future studies should aim at real-time analysis directly after collection, in order to avoid potential VOC alterations based on differences in sampling and storing methods. However, in both research and clinical settings, this will entail logistic challenges.

Outcome of comparison between VOCs deriving from rectal swabs and fecal samples in the present study differ from a similar experiment on microbiota profiling [46]. Microbiota composition on fecal samples and rectal swabs profiles seemed to be comparable [46], whereas we observed significant differences in VOC profiles between the two niches. Possibly, VOCs deriving from the swab itself may interact with the eNose sensors, influencing the measured VOC profile, thereby explaining the observed differences [47]. However, the observation that rectal swab profiles differ from empty swab profiles indicates that rectal swabs could be of value in clinical diagnostics, since a signal different from an empty swab was obtained. Hypothetically, rectal swabs could therefore serve as interesting tool for VOC profiling in diagnostics and/or follow-up of diseases of the distal colon, like ulcerative colitis and diverticulitis. Advantage of swabs over fecal samples are that swabs can be obtained easily at set times and overcome the everlasting stigma about feces collection present in Western world, preventing from adequate sampling by subjects [48].

Our study demonstrated that center of birth has a significant influence on fecal VOC profiles of preterm infants. Obviously, differences in diet, medication use and bacterial exposure between hospitals may all have contributed to observed differences. Moreover, inter-individual variation of these factors in neonates born at the same hospital also occurs, explaining the suboptimal accuracy values obtained in this study. Present study results are consistent with data available from studies comparing exhaled breath samples obtained from an international cohort [49,50]. However, since studies on the influence of birth center on fecal VOCs are lacking, we here focus on comparison with reported microbiota studies. Microbial studies have demonstrated similar results to our observations; center of birth seems to correlate with a distinct intestinal microbiota colonization pattern [51,52,53,54]. Caution should therefore be taken regarding the generalizability of studies about microbiota and consequently fecal VOCs as biomarker for diseases. Research about the potential of fecal VOCs as biomarker in a specific disease should ideally be conducted in large trials, comprising different geographic locations and covering an extended period of time to minimize the risk of bias.

Our study has one important limitation. Since Cyranose320^®^ outcomes are presented as alterations in electrical resistance instead of presence and abundance of individual VOCs, our results do not allow for provision of an optimized sampling and storing method for fecal VOC analysis by means of an eNose. In addition, since different types of eNose devices exhibit a wide diversity of sensor types, results cannot be readily extrapolated from one type of eNose to another type of eNose, thereby challenging eNose implementation in clinical setting [2].

However, this is the first study to assess the effect of clinically crucial variables on VOC profiles obtained by an eNose, by using a large number of fecal samples. Simultaneously, all other possible confounding factors were retained the same, ensuring optimal comparison based on the variable of interest. This study highlights the urge to standardize methodology in research setting, since all studied variables appear to influence fecal VOC profile. Future studies should focus on the identification of disease-specific VOCs by means of GC-MS in a standardized manner. With detection of these individual, disease specific VOCs, a tailored eNose should be developed, allowing for accurate detection of the disease of interest. Combining both techniques bears an additional advantage. Since an eNose needs to be trained by building a large and representative database before it can classify unknown samples, focusing solely on disease- specific VOCs would make a large initial database redundant [19]. Since application of a tailored eNose in clinical practice needs to accurately detect clinical conditions under non-standardized circumstances, as this is not always feasible in clinical practice, disease specific VOCs with optimal accuracy are required.

To conclude, a broad range of sampling conditions and environmental factors seem to have a significant influence on detected fecal VOC profiles, as measured by an eNose device. However, we firmly believe that analysis of fecal VOCs may hold promise as a diagnostic tool, although different methodological challenges need to be overcome. These observations underline the need for standardization of methodology, by developing standard operating procedures, prior to application of eNose technology in clinical practice, and should be taken into account while interpreting and comparing different study outcomes. Since fecal VOC profiles of clinical patients are influenced by local protocols, future research should concentrate on the detection of disease-specific VOCs, to limit the risk for bias by environmental factors.

## Figures and Tables

**Table 1:** Principle component analysis per variable of interest with corresponding *p*-values.

	PC 1 (*p*-Value)	PC 2 (*p*-Value)	PC 3 (*p*-Value)	PC 4 (*p*-Value)
Sample mass (g)				
0.2 vs. 0.5	*0.036*	0.841	*0.016*	0.581
0.2 vs. 2.0	*<0.001*	*<0.001*	*0.020*	0.220
0.5 vs. 2.0	*<0.001*	*<0.001*	0.937	0.077
Number of freeze–thaw cycles (−20 °C—room temperature)				
Fresh feces vs. 1 cycle	*<0.001*	*<0.001*	*<0.001*	*0.018*
Fresh feces vs. 3 cycles	0.641	*0.001*	*<0.001*	*<0.001*
1 cycle vs. 3 cycle	*0.001*	*0.003*	*<0.001*	*<0.001*
Sample temperature (°C)				
4 vs. 21	*<0.001*	0.141	0.824	0.916
4 vs. 37	*<0.001*	0.521	0.054	*0.001*
21 vs. 37	*0.001*	*0.035*	0.085	*0.001*
Water content (Feces (g):H_2_O (mL))				
1:0 vs. 1:1	0.089	*0.017*	0.167	0.106
1:0 vs. 1:2	*<0.001*	*<0.001*	0.072	0.408
1:0 vs. 1:5	*0.001*	*<0.001*	0.093	0.198
1:1 vs. 1:2	*0.028*	*0.001*	0.673	0.429
1:1 vs. 1:5	0.069	*0.002*	0.764	0.742
1:2 vs. 1:5	0.702	0.825	0.903	0.643
Duration of storage at room temperature				
Fresh feces vs. 3 h storage	*<0.001*	*0.001*	*<0.001*	0.718
Comparison rectal swab and fecal sample				
Fecal sample vs. Rectal swab	*<0.001*	*0.018*	*0.002*	0.103
Fecal sample vs. Empty swab	*<0.001*	0.406	0.458	0.362
Rectal swab vs. Empty swab	*<0.001*	0.112	*0.015*	*0.013*
Center of origin				
AMC vs. VUmc	*0.079*	*0.003*	0.109	0.598
AMC vs. MMC	0.480	*<0.001*	0.135	0.050
VUmc vs. MMC	*0.031*	0.881	0.774	*0.043*

**Table 2:** Performance characteristics with corresponding sensitivity, specificity and accuracy values of fecal VOC-analysis for each variable of interest were obtained after internal validation.

	Sensitivity (%)	Specificity (%)	Overall Accuracy (%)
Sample mass (g)			
0.2	60.0	55.1	
0.5	52.7	58.0	
2.0	81.6	80.0	64.4
Number of freeze–thaw cycles (−20 °C—room temperature)			
Fresh feces	100.0	95.7	
1 cycle	87.2	82.0	
3 cycles	83.6	92.0	89.7
Sample temperature (°C)			
4	100.0	93.9	
21	71.4	70.0	
37	66.0	71.4	78.4
Water content (Feces (g):H_2_O (mL))			
1:0	51.5	68.0	
1:1	29.7	22.4	
1:2	36.6	52.0	
1:5	20.0	10.0	38.1
Duration of storage at room temperature			
Fresh feces	100.0	100.0	
3 h storage	100.0	100.0	100.0
Comparison rectal swab and fecal sample			
Fecal sample	78.9	68.2	
Rectal swab	66.7	75.0	
Empty swab	92.0	92.0	78.9
Center of origin			
AMC	56.4	41.3	
VUmc	23.0	32.5	
MMC	34.8	41.3	39.6

## Supplementary Materials

Supplementary File 1

## Abbreviations

The following abbreviations are used in this manuscript:

AMC	Academic medical center
eNose	electronic nose
GC-MS	gas chromatography- mass spectrometry
MMC	Maxima Medical Center
PC	principle component
VOC	volatile organic compound
VUmc	Vrije Universiteit medical center
