# Supplementary material for: Effects of Sampling Conditions and Environmental Factors on Fecal Volatile Organic Compound Analysis by an Electronic Nose Device
Source: Sensors (Basel). 2016 Nov 23;16(11):1967. doi: 10.3390/s16111967 (PMC5134625; doi:10.3390/s16111967)
Supplement: Supplementary File 1 [file sensors-16-01967-s001.pdf]

Supplementary Materials: Effects of Sampling Conditions and Environmental Factors on Fecal Volatile Organic Compound Analysis by an Electronic Nose Device

Daniel J. C. Berkhout, Marc A. Benninga, Ruby M. van Stein, Paul Brinkman, Hendrik J. Niemarkt, Nanne K. H. de Boer and Tim G. J. de Meij

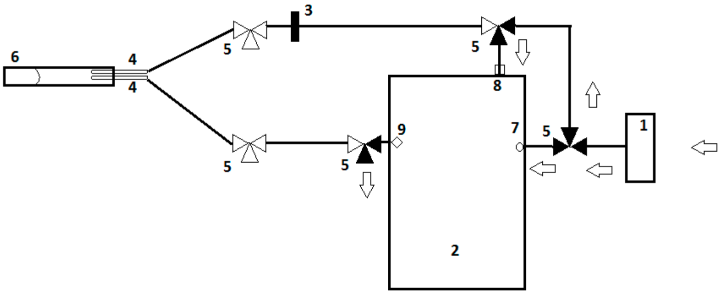

(**a**)

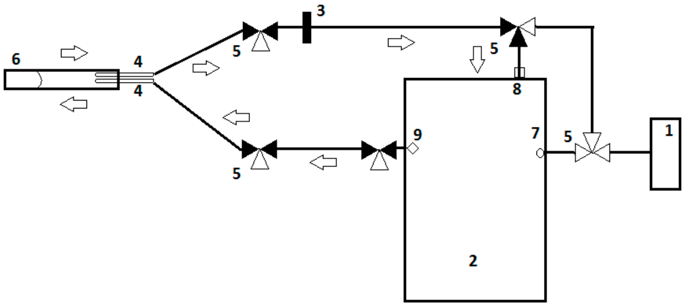

(**b**)

Figure S1. A schematic illustration of electronic nose setup during: (**a**) purging of the sensors and subsequently obtaining a baseline reference signal; and (**b**) performing the actual sample measurement. A dark cone in a three-way valve (*number 5*) illustrates an air flow can pass, while a white cone prevents this. The arrows depict the air flow through the measurement setup. (**a**) First, the sensors are purged for 90 s with filtered air derived from ambient air passing the A1-filter. The airflow, containing the residual volatile organic compounds (VOC), detached from the sensors, is expelled through the exhaust port. Subsequently, a baseline reference signal is obtained in 30 s using filtered air; (**b**) After the baseline reference signal is obtained, the actual measurement takes place in 60 s. By rotating several three-way valves, a closed loop in connection with the fecal sample is formed (6). This loop prevents dilution of fecal VOCs with ambient air and moreover, causes a continuously flow of fecal VOC passing the sensors. After the measurement, the three-way valves are rotated back to their original positions (Figure 1a) and the sensors are purged. (1) A1 filter; (2) Cyranose320®; (3) Polyethersulfone filter; (4) blunt needle; (5) three-way valve; (6) a vacutainer containing feces; (7) purge inlet; (8) sensor inlet; (9) exhaust portal; (10) oxygen hose.

Table S1. Analyzed subjects and samples per center.

	Number of Subjects (*n*)	Number of Samples (*n*)
**AMC**	34	658
**VUmc**	16	245
**MMC**	21	382

Abbreviations: AMC = Academic medical center; MMC = Maxima medical center; VUmc = Vrije Universiteit medical center.

Table S2. Principal components per variable of interest differing three or more standard deviations from the mean.

	**PC1**	**PC2**	**PC3**	**PC4**
Sample mass (g)				
**0.2**	1	-	-	-
**2.0**	-	-	-	1
Number of freeze-thaw cycles (−20 °C—room temperature)				
**Fresh feces**	2	-	2	-
Sample temperature (°C)				
**4**	-	-	1	1
**37**	-	-	-	1
Water content (Feces (g):H_2O (mL))				
**1:1**	-	1	-	-
Duration of storage at room temperature				
**Fresh feces**	1	-	1	-
Rectal swab sampling				
**Fecal sample**	-	1	2	-
**Rectal swab**	-	-	-	1
**Empty swab**	-	-	-	-
Center of origin				
**AMC**	9	4	6	3
**VUmc**	1	-	-	1
**MMC**	5	2	4	1

Abbreviations: AMC = Academic medical center; MMC = Maxima Medical Center; VUmc = Vrije Universiteit medical center.
